# Analgesic effect of local anaesthetic in haemorrhoid banding: systematic review and meta-analysis

**DOI:** 10.1007/s00384-024-04609-8

**Published:** 2024-03-04

**Authors:** Eleanor G. R. Watson, Hwa Ian Ong, Nicholas J. W. Shearer, Philip J. Smart, Adele N. Burgess, David M. Proud, Helen M. Mohan

**Affiliations:** 1 Faculty of Medicine, Dentistry and Health Sciences, University of Melbourne, Melbourne, VIC Australia; 2 Department of Surgery, Austin Hospital, Melbourne, VIC Australia; 3 Department of Anaesthesia, Royal Melbourne Hospital, Melbourne, VIC Australia

**Keywords:** Haemorrhoids, Anaesthesia, Local, Pain management, Surgeons

## Abstract

**Purpose:**

Rubber band ligation of haemorrhoids can be,painful and there is no consensus regarding the optimal analgesic strategy. This study aims to determine whether there is a difference in post-procedural pain in adults undergoing haemorrhoid banding who have received local anaesthetic, a pudendal nerve block or no regional or local analgesia.

**Methods:**

MEDLINE, Embase, Google Scholar and clinical trial registries were searched for randomised trials of local anaesthetic or pudendal nerve block use in banding. Primary outcomes were patient-reported pain scores. The quality of the evidence was assessed using the GRADE approach.

**Results:**

Seven studies were included in the final review. No articles were identified that studied pudendal nerve blocks. The difference in numerical pain scores between treatment groups favoured the local anaesthetic group at all timepoints. The mean difference in scores on a 10-point scale was at 1 h,—1.43 (95% CI—2.30 to—0.56, *p* < 0.01, *n* = 342 (175 in treatment group)); 6 h,—0.52 (95% CI—1.04 to 0.01, *p* = 0.05, *n* = 250 (130 in treatment group)); and 24 h,—0.31 (95% CI—0.82 to 0.19, *p* = 0.86, *n* = 247 (127 in treatment group)). Of reported safety outcomes, vasovagal symptoms proceeded to meta-analysis, with a risk ratio of 1.01 (95% CI 0.64–1.60). The quality of the evidence was rated down to ‘low’ due to inconsistency and imprecision.

**Conclusion:**

This review supports the use of LA for reducing early post-procedural pain following haemorrhoid banding. The evidence was limited by small sample sizes and substantial heterogeneity across studies.

**Registration:**

PROSPERO (ID CRD42022322234)

**Supplementary Information:**

The online version contains supplementary material available at 10.1007/s00384-024-04609-8.

## Introduction

Haemorrhoids are the most common anorectal condition, affecting up to 40% of the population in Western countries [1]. Conservative management options for symptomatic haemorrhoids include dietary modifications, glyceryl trinitrate, nifedipine and topical preparations containing steroids and anaesthetics [2, 3]. Failing this, non-operative treatments for Grade I–III haemorrhoids include rubber band ligation (RBL), sclerotherapy and infrared coagulation [2]. Of these options, RBL is the most common [4], as it is effective, comparatively simple to perform, associated with minimal complications and can be done as a day procedure in the outpatient setting [2, 5, 6].

Rubber band ligation involves placing a band on the mucosal aspect of an internal haemorrhoid, which reduces blood flow and causes necrosis and sloughing of the tissue. In theory, the procedure should cause minimal-to-no pain because bands are placed above the dentate line. Yet, pain is the most common complication of RBL, affecting up to 90% of patients and typically worst in the first 24 h post procedure [6–10]. There is a need to better control pain in these patients in the interest of reducing post-operative opioid use and time-to-discharge and improving patient quality of life.

Currently, there is a lack of evidence around the best analgesic strategy in patients undergoing RBL. In practice, patients may receive a submucosal injection of local anaesthetic (LA) at the banding site, a pudendal nerve block (PNB) or no local or regional analgesia, with the choice of approach proceduralist dependent. In 2015, Sajid et al. performed a systematic review and meta-analysis finding that post-procedural pain scores were significantly lower in patients undergoing RBL of haemorrhoids with submucosal LA injection than patients with no LA [8]. The review of four randomised controlled trials did not look at surrogate measures of post-procedural pain such as analgesia use and time-to-discharge and was limited by the small sample sizes and heterogeneity of included trials. Additionally, although several meta-analyses have studied the efficacy of PNB in haemorrhoidectomies [11, 12], no reviews have included participants who have received a PNB for RBL. There is a need for high-quality research to standardize and optimise analgesia in patients undergoing this common procedure. The aim of this systematic review was to determine the effect of LA vs PNB vs no regional or local analgesia on post-procedural pain.

## Methods

This systematic review and meta-analysis has been written according to the Preferred Reporting Items for Systematic Reviews and Meta-Analyses (PRISMA) guidelines [13]. No ethical approval or consent were required, and the review was prospectively registered with PROSPERO (ID CRD42022322234).

### Search strategy

A literature search of MEDLINE (Ovid) and Embase (Ovid) was performed by two researchers (EW and NS, May 2022). The full search conducted in MEDLINE is shown in Online Resource 1. Hand searches of the reference lists of included studies, Google Scholar and clinical trial registries (Australian New Zealand Clinical Trials Registry, ClinicalTrials.gov and European Union Clinical Trials Register) were also performed to find relevant articles not yet identified in the database search. Searches were not restricted by language or publication date.

### Eligibility criteria

For inclusion in this review, studies met the following criteria: (1) randomised controlled trial; (2) studied adult participants who underwent RBL of haemorrhoids; (3) intervention participants received LA or PNB, and control participants received no local or regional analgesia; and (4) measured pain as an outcome, either via a pain score or a surrogate marker of pain (e.g. post-operative analgesia use). Participants were included irrespective of prior banding procedures and technique of banding and LA administration. The review was restricted to randomised trials due to the potential for confounding with respect to patient selection for the administration of LA/PNB or neither.

This review excluded studies where participants underwent RBL of haemorrhoids in conjunction with another major anorectal procedure (e.g. fistulectomy) or had a comorbid anorectal condition (e.g. Crohn’s disease). Participants were still included if they underwent a concurrent minor interventional procedure (e.g. polypectomy) or non-interventional procedure (e.g. diagnostic colonoscopy).

### Study selection and data management

Studies identified through the literature search were uploaded to Covidence systematic review software (Veritas Health Innovation Ltd, Melbourne) for screening. After duplicate removal, study titles and abstracts were assessed against the eligibility criteria by two independent reviewers (EW and NS), with relevant articles subsequently assessed in full text. Discrepancies in reviewers’ selected studies were resolved by a third investigator (HM).

Data was extracted from the final set of included studies using a pre-defined data collection form in Microsoft Excel (Microsoft Corporation, Washington). Data extraction was performed independently by two reviewers (EW and NS), with discrepancies in the data reviewed by investigators together and a third investigator (HM) available for dispute resolution when required.

### Data items and outcomes

Reviewers collected the following data on study characteristics: author; year; publication language; study country; follow-up period; method of control; method of intervention; and scale used to measure pain scores. To determine whether treatment groups were balanced between studies with respect to important variables, reviewers collected baseline participant data (age, sex, history of prior banding procedures, presence of pre-operative anorectal pain, regular analgesia use) and procedural methods (number of haemorrhoids banded, highest grade of haemorrhoid banded and intraoperative analgesia administered).

Post-procedural patient-reported pain scores at 1, 6 and 24 h were considered the primary end point. Secondary end points were time-to-discharge and post-procedural analgesia consumption. Post-procedural complications were also assessed.

### Data synthesis and statistical analysis

Raw data for each study were recorded using the same measures reported in original manuscripts. All analysis was undertaken in R Studio using the *meta* package [14, 15]. Of the seven studies identified in the systematic review, five reported post-procedure pain using an analogue scale and were included in the meta-analysis. The two studies that reported a categorical pain outcome were not included in this analysis. For the complication outcome, vasovagal episodes were reported in a homogenous manner in four studies and thus proceeded to meta-analysis.

To allow comparison between post-procedure pain results, all reported measures were converted to a mean with standard deviation. In cases where median and interquartile range were reported, a method of estimation proposed by Wan et al. was employed for the conversion [16]. In the case of Hooker et al., the analogue pain scale was reported out of 100, and therefore results were divided by 10. Where studies did not report pain at one of the pre-specified timepoints (1, 6 and 24 h), the pain score collected closest to the timepoint was used. All studies had a non-placebo control group that was used for the analyses.

For the meta-analyses, random effects models were used in all analyses. Where continuous data were available, mean difference (MD) with a corresponding 95% CI were reported, with inverse variance weighting used for the pooled results. Where categorical data were available, the Mantel–Haenszel method was used, and if a study group had no outcomes to occur, a value of 0.5 was inserted into the cell.

For the primary end point, a minimum reduction in pain score of one point on a 10-point scale (MD of -1.00) was considered a clinically significant result. It has previously been shown in patients recovering from surgery that a change of 10 on a 100-point Visual Analogue Scale signifies clinically meaningful improvement [17].

### Quality assessment

Two researchers (EW, NS) independently assessed the quality of included trials using the Cochrane Collaboration’s revised risk-of-bias tool for randomised trials (RoB 2) of parallel or crossover design [18]. Heterogeneity was assessed using the I^2^ statistic and categorised according to definitions described by Cochrane [19]. The overall quality of the cumulative evidence for the primary outcome was assessed using the Grading of Recommendations, Assessment, Development and Evaluations (GRADE) approach [20].

## Results

### Literature search

**Fig. 1:**
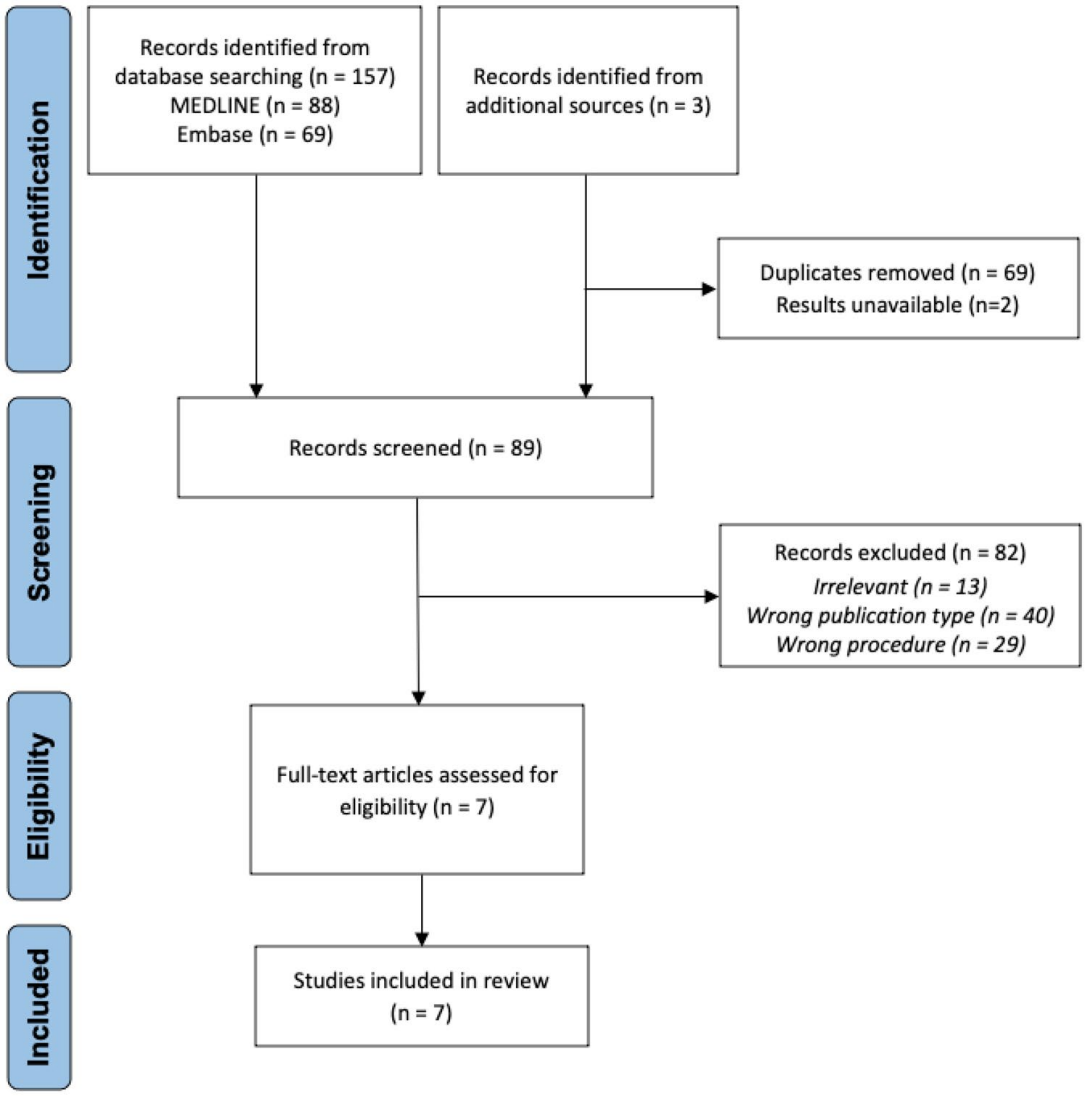
Flow chart of the literature search according to PRISMA guidelines

The literature search in MEDLINE (Ovid) and Embase (Ovid) returned 157 articles. One additional article was included from Google Scholar. Two additional relevant studies were identified through searches of ClinicalTrials.gov (ID NCT03797703 and NCT02130830), but these were not included as study results were not publicly available and were unable to be obtained by authors upon request. Sixty-nine duplicates were removed, and 82 records were excluded on title and abstract screening. Seven records were assessed in full text for eligibility, and all of these were included in the final analysis (Fig. 1).

### Study characteristics

All studies provided information on study design and methods (Online Resource 2). All studies were parallel randomised controlled trials except one, which was a crossover trial [21]. The search identified no studies that used PNB for analgesia in haemorrhoid banding. In all studies, the intervention was LA at the area of the band: Four studies used bupivacaine injection [7, 22–24], one used lignocaine injection [10], one used lignocaine topical gel [25] and one used a cinchocaine-containing suppository [21]. Five studies used a non-placebo control [10, 22–25], and two studies used a placebo [7, 21] in addition or instead. The follow-up period for assessment of pain outcomes ranged from 2 h to 6 weeks. Five studies measured pain on a numerical scale: Four used a 10-point visual analogue scale [22–25] and one used a 100-point linear analogue scale [7]. The other two studies used a 4-point categorical scale [10] and a binary assessment (pain/no pain) [21].

### Participant characteristics

The total number of participants in included studies was 545. Of the reported participant characteristics, none varied significantly between control and intervention groups within or across studies (Table 1). Across the five studies that reported participant age and gender [7, 22–25], the mean age of participants in treatment groups was 37–56. Two studies reported pre-procedural pain and prior banding [24, 25]; 18–35% of participants experienced pain at baseline, and 8–17% of participants had undergone banding previously. Across the five studies that reported the number of bands applied intraoperatively [10, 22–25], most participants had > 1 band applied (70–100% of participants). No studies reported on participants’ regular analgesia use or specified whether any additional analgesia was administered intraoperatively (i.e. by the anaesthetist). One study reported the degree of haemorrhoids banded in study groups [22].

**Table 1:** Summary of participant characteristics in included trials

	**Age (mean)**		**Sex (% female)**		**Participants with pain before procedure (%)**		**Participants with a history of haemorrhoid procedures (%)**		**Participants who had > 1 band applied (%)**	
First author	NLA	LA	NLA	LA	NLA	LA	NLA	LA	NLA	LA
Hooker	53.3 (P), 50.7 (NP)	50.50	45 (P), 42 (NP)	38.10	-	-	-	-	-	-
Baloch	40.23	37.33	46.67	23.33	-	-	-	-	73.33	70.00
Gokalp	52*	56*	29.17	32.86	-	-	-	-	100.00	100.00
Kwok	54*	51*	37.50	25.00	31.25	35.00	15.63	7.50	97.00	90.00
Law	-	-	-	-	-	-	-	-	100.00	100.00
Sharma	54.00	56.00	41.18	55.56	17.65	22.22	11.76	16.67	100.00	94.44
Williams	-	-	-	-	-	-	-	-	-	-

*P* placebo, *NP* non-placebo, *LA* local anaesthetic, *NLA* no local anaesthetic. ‘-’ denotes data not reported by study

*Median instead of mean

### Primary outcomes

**Fig. 2:**
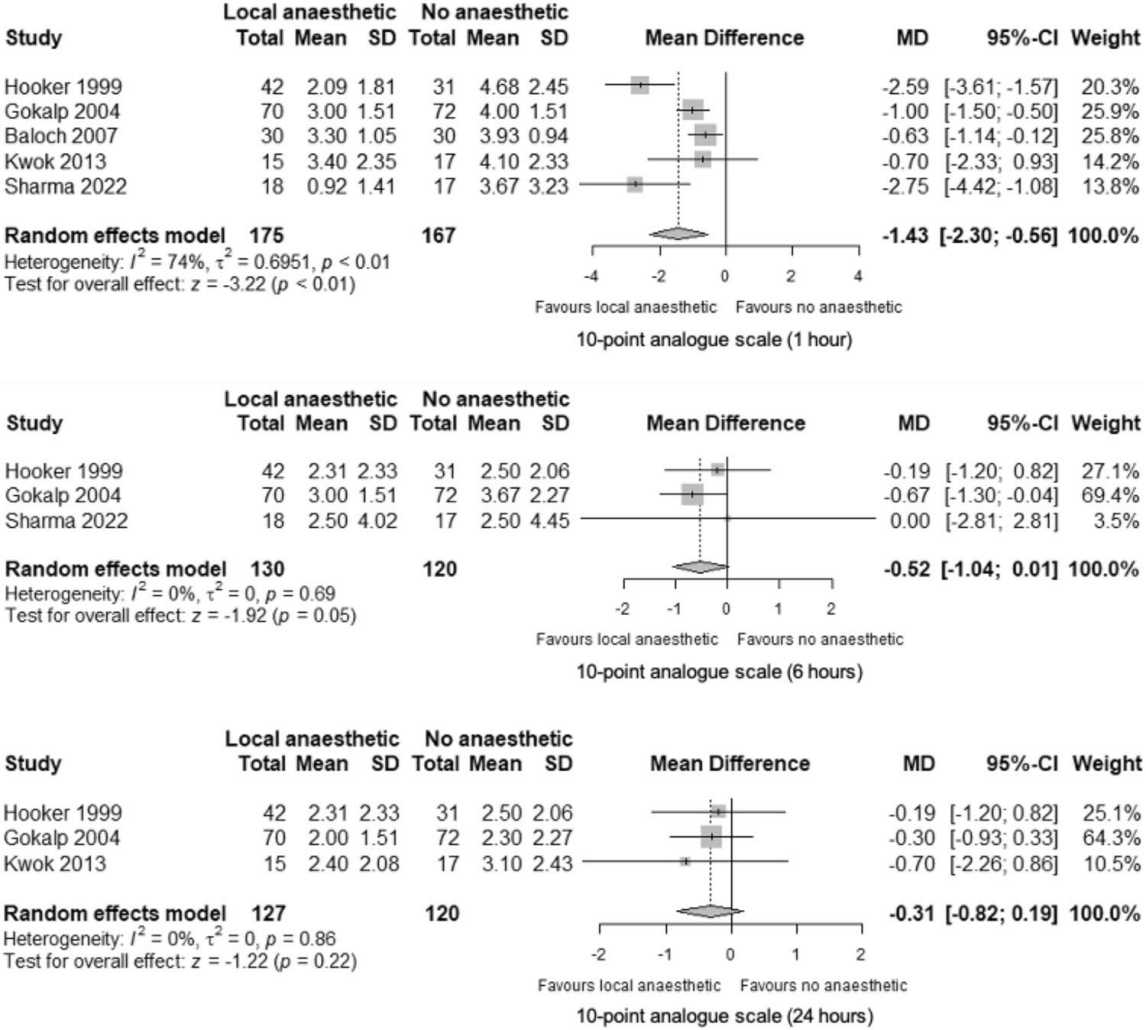
Mean difference in pain scores between participants undergoing rubber band ligation with local anaesthetic or no local anaesthetic at 1 h, 6 h and 24 h post-procedure

Regarding the primary outcome of post-procedural pain scores at 1, 6 and 24 h, the results were in favour of LA at all timepoints, and the difference between groups was significant at one hour. Overall, the mean difference in post-procedural pain scores between.

study groups decreased over time (Fig. 2).

A total of 342 participants were included in meta-analysis of the primary outcome at one hour post-procedure, of whom 175 received LA and 167 did not receive LA [7, 21–24]. At 1 h, the mean difference in patient-reported pain scores was -1.43 points on a 10-point analogue scale (95% CI -2.30 to -0.56, *p* < 0.01), favouring LA (Fig. 2). Mean pain scores ranged from 0.92 to 3.40 and 3.67 to 4.68 in the LA and no LA groups, respectively.

At 6 h post-procedure, 250 participants were included in the analysis (130 received LA, 120 did not) [7, 23, 25]. The mean difference in patient-reported pain scores at 6 h was -0.52 (95% CI -1.04 to 0.01, *p* = 0.05), favouring LA (Fig. 2). Mean pain scores ranged from 2.31 to 3.00 and 2.50 to 3.67 in the LA and no LA groups, respectively.

At 24 h post-procedure, 247 participants were included in the analysis (127 received LA, 120 did not) [7, 23, 24]. The mean difference in patient-reported pain scores at 24 h was -0.31 (95% CI -0.82 to 0.19, *p* = 0.86), favouring LA (Fig. 2). Mean pain scores ranged from 2.00 to 2.40 and 2.30 to 3.10 in the LA and no LA groups, respectively.

Two studies did not measure pain on a numerical scale and were therefore excluded from meta-analysis. Williams et al. reported the proportion of participants who had pain 24 h post procedure; 56% had pain in the no LA group vs 63% in the LA group [21]. Law et al. reported the proportion of participants who reported ‘no pain’, ‘slight pain’, ‘moderate pain’ and ‘severe pain’ over the 2 weeks following their RBL procedure [10]. Overall, 63% of participants in the LA group experienced slight or moderate pain compared to 49% of participants in the no LA group. No participants reported severe pain.

### Secondary outcomes

The only surrogate marker of pain reported by studies was post-procedural analgesia use. Three studies reported the mean number of analgesia tablets used [10, 21, 23], with participants in the no LA group using 1.75–2.70 tablets, and participants in the LA group using 1.47–2.40 tablets. One study reported the proportion of participants who had new analgesia use 72 h post-procedure [25], with 7/17 participants in the no LA group and 9/18 participants in the LA group commencing analgesia in this time. No studies reported time-to-discharge post-procedurally.

### Safety data

**Fig. 3:**
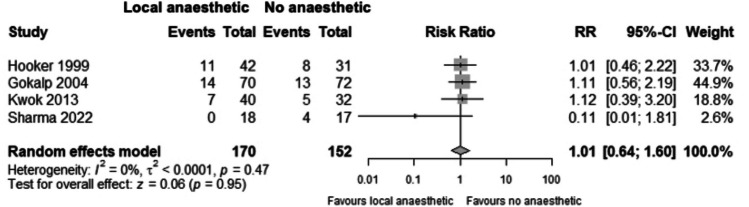
The risk of vasovagal symptoms in participants undergoing rubber band ligation of haemorrhoids with local anaesthetic and no local anaesthetic

Post-procedural complications were reported by all studies except Williams et al. (Tab.2). Studies did not report the number of patients in each group that had at least one complication, precluding meta-analysis of overall complications. Four studies reported the overall incidence of rectal bleeding (4–53%) [7, 10, 22, 23] and vasovagal symptoms (11–40%) [7, 23–25], and two reported the overall incidence of infection/sepsis (0% and 0%) [10, 23], urinary retention (8% and 29%) [7, 23], nausea (9% and 32%) [7, 23] and tenesmus (25% and 1%) [10, 23]. Some studies reported outcomes by treatment group, with the incidence of rectal bleeding and tenesmus similar between groups; the incidence of urinary retention higher in intervention groups; and the comparative incidence of nausea varying across studies. Meta-analysis was undertaken for the incidence of vasovagal symptoms, with a risk ratio of 1.01 (95% CI 0.64–1.60) (Fig. 3).

**Table 2:** The incidence of complications following rubber band ligation of haemorrhoids with and without local anaesthetic

**Post-procedural complications reported as: number (proportion)**						
**First author**	**Rectal** **bleeding**	**Infection/sepsis**	**Urinary** **retention**	**Vasovagal** **symptoms**	**Nausea**	**Tenesmus**
Baloch (*n* = *60*)						
Control	-	-	-	-	-	-
Intervention	-	-	-	-	-	-
Total	7 (12%)	-	-	-	-	-
Gokalp (*n* = *142*)						
Control	10 (14%)	0 (0%)	6 (8%)	13 (18%)	6 (8%)	19 (26%)
Intervention	9 (13%)	0 (0%)	5 (16%)	14 (20%)	7 (10%)	16 (23%)
Total	19 (13%)	0 (0%)	11 (8%)	27 (19%)	13 (9%)	35 (25%)
Hooker (*n* = *73*)						
Control	16 (52%)	-	8 (26%)	14 (45%)	13 (42%)	-
Intervention	23 (55%)	-	13 (31%)	15 (36%)	10 (24%)	-
Total	39 (53%)	-	21 (29%)	29 (40%)	23 (32%)	-
Kwok (*n* = *72*)						
Control	-	-	-	5 (16%)	-	-
Intervention	-	-	-	7 (18%)	-	-
Total	-	-	-	12 (17%)	-	-
Law (*n* = *101*)						
Control	-	0 (0%)	-	-	-	-
Intervention	-	0 (0%)	-	-	-	-
Total	4 (4%)	0 (0%)	-	-	-	1 (1%)
Sharma (*n* = *35*)						
Control	-	-	-	4 (24%)	-	-
Intervention	-	-	-	0 (0%)	-	-
Total	-	-	-	4 (11%)	-	-

‘-’ denotes data not reported by study. Hooker et al. used both placebo and non-placebo control groups; only data for non-placebo group is shown. Williams et al. did not report any safety data and is therefore not included in the table

### Quality assessment and risk of bias

In our GRADE assessment of the primary outcome (pain scores), the quality of the cumulative evidence was rated down to ‘low’ based on inconsistency and imprecision (Online Resource 3). There was substantial heterogeneity in the pain scores of included studies: At 1 h, I^2^ was 74% (50–90%), and at 6 and 24 h, *I*^2^ was measured as 0% due to the difference crossing zero. Imprecision was rated as ‘serious’ or ‘very serious’ at all timepoints because of small sample sizes and wide confidence intervals. Using the RoB 2 tools for parallel and crossover trials, the overall risk of bias with respect to the primary outcome in both parallel [7, 10, 22–25] and crossover [21] studies was rated as ‘some concerns’, mostly pertaining to ‘selection of the reported result’ and ‘measurement of the outcome’, which was at times unclear but not serious enough to further downgrade the evidence (Online Resource 4). Only one study included a funding statement [24], with no funding declared.

## Discussion

This systematic review and meta-analysis support the use of LA in rubber band ligation of haemorrhoids. Local anaesthetic had a statistically significant effect on post-procedural pain early in the recovery period. Analysis of the effect of LA on post-procedural analgesia consumption and time-to-discharge were limited by a lack of data. The quality of the evidence was downgraded to ‘low’, largely due to small sample sizes, but the direction of treatment effect was consistent across studies reporting numerical pain scores. Herein lies the impetus for robust, well-designed research to contribute high-quality data to this area of enquiry.

This was the first systematic review of analgesia in rubber band ligation to include PNB in the search strategy, and no eligible studies were identified. The search returned multiple studies of PNB in haemorrhoidectomies, suggesting that the search scope was adequate and that this represents a true gap in the literature. While our original research question may have been best analysed using a network meta-analysis, an absence of PNB studies precludes this approach. This review identified two articles not included in the previous 2015 review [8], published in 2022 and 1972. The latter was likely not included as the intervention involved a suppository containing LA, rather than an injection of anaesthetic, although the previous review eligibility criteria did not specify the route of LA administration. Although our review included grey literature and all publication dates and languages in the search strategy, publication bias remains possible, particularly as data from trials identified in the registry search were unable to be obtained.

The previous 2015 review included four randomised trials, only two of which were included in the meta-analysis of post-procedural pain scores [8]. One study was excluded because pain was not measured using a numerical scale, but it is unclear why the second study was excluded from the analysis. The present study adds to the previous review by including seven randomised trials, five of which were included in the meta-analysis of post-procedural pain scores (five at 1 h, three at 6 h and three at 24 h). The previous review only examined mean pain score within 6 h of the procedure, whereas our analysis includes pain scores at multiple discrete timepoints including 1 h, when the maximal effect of the intervention was observed. Accordingly, the previous review possibly underestimated the treatment effect of LA.

The greatest difference in participant pain scores at 1 h post-procedure can be explained by the duration of action of the anaesthetic agents. The most common LA agent used was bupivacaine, followed by lignocaine and cinchocaine. The duration of action of these agents is 2–8 h, 1–2 h and 2–3 h, respectively [26]. It is therefore not surprising that the greatest difference in pain scores amongst participants was at 1 h post-procedure, with a declining effect over time and no effect at 24 h, although the known trend towards lower reductions in pain at later timepoints due to underlying differences in baseline risk should also be considered [27].

Our ability to interpret safety outcome data was limited by inconsistent reporting and possible reporting bias. Only vasovagal symptoms were homogenously reported across studies and amenable to meta-analysis. The highest incidence of complications was reported for rectal bleeding, affecting more than half of patients in both control and intervention groups in one study [7]. Reporting the degree of rectal bleeding would be useful in this context as minor bleeding is expected following RBL, with the passage of banded haemorrhoidal tissue, and as such, it is possible that other studies did not consider this a complication. The only outcome with a consistently higher incidence in intervention groups was urinary retention, reported by two studies, both of which used bupivacaine [7, 23]. A recent meta-analysis of PNB in haemorrhoidectomies reported a lower incidence of urinary retention in participants receiving LA [12], although most studies used ropivacaine, which has a favourable safety profile over bupivacaine [28]. Life-threatening complications including systemic LA toxicity were never reported in our literature search.

The pain scores reported in this study not only support the use of LA but confirm that rubber band ligation causes pain. This challenges the longstanding belief that haemorrhoid banding is painless as it is only performed on haemorrhoids above the dentate line. The reason for pain following the procedure has not been investigated, but possible explanations include incorrect band placement below the dentate line; spasm of the anal sphincter muscles; patients reporting visceral sensations of distension or dragging as pain; and the relatively gradual transition from somatic to autonomic innervation at the dentate line—described by anatomist Last [29]—meaning that somatic nerves are still stimulated by the procedure.

We consider the measured improvement in post-procedural pain of 1.43 points at 1 h to be clinically significant. Aside from the clear benefit to patients in improving postoperative experience and recovery, this result has potential cost and health system implications. Banding is usually performed as a day procedure in high-turnover lists treating large numbers of patients. Improving post-procedural pain is central to facilitating timely and independent discharge home from labour-intensive, costly theatre recovery units. With an intervention that is inexpensive and quick to perform, future randomised trials should include a cost–benefit analysis. Greater improvements in post-procedural pain may also be realised when the cause of pain is better understood. Targeting possible spasm of the anal sphincter using topical calcium channel blockers, for example, warrants investigation.

There was variation in the outcome measures reported by studies, limiting the extent of meta-analysis. It was not clear why some studies chose to use unvalidated categorical outcome measures of pain, or why pain was measured at particular timepoints. Secondary outcomes were reported by few or no studies (analgesia consumption, time-to-discharge). Interestingly, in the two studies that reported pain using categorical scales [10, 21], the direction of treatment effect opposed the meta-analysis of studies of numerical pain scores, with a marginally higher proportion of participants who received LA reporting pain than patients without LA. To standardise outcome reporting and strengthen future analyses, studies should consider the use of Patient Reported Outcome Measures (PROM) Core Outcome Sets (COS). A COS for haemorrhoid disease has recently been developed in collaboration with the European Society of Coloproctology, with primary outcomes including symptoms such as pain, prolapse and itch and secondary outcomes including complications, haemorrhoid recurrence and patient satisfaction [30]. Nonetheless, this COS is related to the treatment of haemorrhoidal disease, not analgesic interventions supporting these treatments, limiting its relevance to the topic of this review. A COS for analgesia in proctology would be valuable.

A further limitation of this review was methodological variation in the interventions of included studies. Given the relative paucity of literature around the topic of this review, the eligibility criteria were intentionally broad, resulting in differences in LA route of administration, anaesthetic type and additional agents (e.g. adrenaline, hydrocortisone), all of which could be expected to alter the pharmacologic properties of the intervention. Another possible source of heterogeneity in study outcomes was the presence of additional procedures or interventions. Although the eligibility criteria excluded participants undergoing major anorectal procedures, no studies specified whether any concurrent procedures were performed outside of diagnostic imaging (endoscopy). All studies except one [21] specified exclusion of patients with other anorectal conditions, so it is reasonable to assume that no participants undergoing major additional procedures were included. It is possible that some participants underwent polypectomy alongside endoscopy, which could have influenced study outcomes. Additionally, the statistical heterogeneity of pain score results may be explained by baseline consumption of systemic analgesic agents, as has been demonstrated in the case of morphine consumption and heterogeneity in meta-analyses of adjuvant analgesics across surgical procedures [27].

We have demonstrated a lack of high-quality evidence in this common area of practice where the potential for gains, however marginal, may be overlooked because of the prevailing surgical dogma surrounding procedural pain. Given that PNB are considered best practice in haemorrhoidectomies and are gaining popularity in RBL, the absence of research supporting their use in banding also represents a significant gap in the literature. A triple-arm randomised trial comparing submucosal LA and PNB for analgesia in haemorrhoid banding is currently underway (ACTRN12622000006741p) [31]. Further research is also needed to determine whether LA influences other clinically relevant measures such as analgesia consumption.

## Supplementary Information

Below is the link to the electronic supplementary material.

Supplementary file1 (PDF 279 KB)

Supplementary file2 (PDF 90 KB)

Supplementary file3 (PDF 126 KB)

Supplementary file4 (PDF 81 KB)

## Data Availability

No datasets were generated or analysed during the current study.
