# Supplementary material for: Analgesic effect of local anaesthetic in haemorrhoid banding: systematic review and meta-analysis
Source: Int J Colorectal Dis. 2024 Mar 4;39(1):34. doi: 10.1007/s00384-024-04609-8 (PMC10912253; doi:10.1007/s00384-024-04609-8)
Supplement: Supplementary file 1 — Supplementary file1 (PDF 279 KB) [file 384_2024_4609_MOESM1_ESM.pdf]

Journal: International Journal of Colorectal Disease

Article title: Analgesic effect of local anaesthetic in haemorrhoid banding: systematic review and meta-analysis

Authors: Eleanor G R Watson[1*], Hwa Ian Ong[2], Nicholas J W Shearer[3], Philip J Smart[2], Adele N Burgess[2], David M Proud[2], Helen M Mohan[2]

[1]University of Melbourne (Faculty of Medicine, Dentistry and Health Sciences), Melbourne (VIC), Australia.

[2]Austin Hospital (Department of Surgery), Melbourne (VIC), Australia.

[3]Royal Melbourne Hospital (Department of Anaesthesia), Melbourne (VIC), Australia.

*Corresponding author

E-mail: egwatson@student.unimelb.edu.au

ORCID: 0000-0003-3614-5132

Twitter: @dreleanorwatson

| # ▲ | Searches | Results |
|---|---|---|
| 1 | Ligation/ | 24430 |
| 2 | ((haemorrhoid* or hemorrhoid* or of hemorrhoid* or of haemorrhoid*) adj3 band*).tw,kf. | 185 |
| 3 | ((haemorrhoid* or hemorrhoid* or band* or rubber band*) adj3 ligation).tw,kf. | 1764 |
| 4 | 1 or 2 or 3 | 25191 |
| 5 | Hemorrhoids/ | 5465 |
| 6 | (hemorrhoid* or haemorrhoid* or piles).tw,kf. | 8418 |
| 7 | 5 or 6 | 9394 |
| 8 | exp Anesthetics, Local/ | 109836 |
| 9 | Anesthesia, Local/ | 18116 |
| 10 | *Nerve Block/ | 15955 |
| 11 | Pudendal Nerve/ | 516 |
| 12 | (pudendal nerve block* or nerve block* or pudendal nerve or regional anesthetic* or regional anaesthetic* or regional analgesia).tw,kf. | 17828 |
| 13 | (anesthesia or anaesthesia or anesthetic* or anaesthetic* or local anesthetic* or local anaesthetic* or local analgesia).tw,kf. | 306469 |
| 14 | (lignocaine or lidocaine or ropivacaine or bupivacaine).tw,kf. | 42440 |
| 15 | 8 or 9 or 10 or 11 or 12 or 13 or 14 | 401236 |
| 16 | 4 and 7 and 15 | 88 |
