# Supplementary material for: Analgesic effect of local anaesthetic in haemorrhoid banding: systematic review and meta-analysis
Source: Int J Colorectal Dis. 2024 Mar 4;39(1):34. doi: 10.1007/s00384-024-04609-8 (PMC10912253; doi:10.1007/s00384-024-04609-8)
Supplement: Supplementary file 2 — Supplementary file2 (PDF 90 KB) [file 384_2024_4609_MOESM2_ESM.pdf]

Journal: International Journal of Colorectal Disease

Article title: Analgesic effect of local anaesthetic in haemorrhoid banding: systematic review and meta-analysis

Authors: Eleanor G R Watson[1*], Hwa Ian Ong[2], Nicholas J W Shearer[3], Philip J Smart[2], Adele N Burgess[2], David M Proud[2], Helen M Mohan[2]

[1]University of Melbourne (Faculty of Medicine, Dentistry and Health Sciences), Melbourne (VIC), Australia.

[2]Austin Hospital (Department of Surgery), Melbourne (VIC), Australia.

[3]Royal Melbourne Hospital (Department of Anaesthesia), Melbourne (VIC), Australia.

*Corresponding author

E-mail: egwatson@student.unimelb.edu.au

ORCID: 0000-0003-3614-5132

Twitter: @dreleanorwatson

First author	Article title	Publication year	Publication language	Country of study	Type of control	Intervention	Blinding	Participant no. (intervention, control)	Follow-up period	Pain scale
Baloch	Effectiveness of Bupivacaine after Rubber Band Ligation of Haemorrhoids for Post Procedue Pain	2007	English	Pakistan	Non-placebo	2mL 0.5% bupivacaine injected into banded tissue after RBL	Double-blind	60 (30, 30)	2 hours	10-point visual analogue scale
Gokalp	A prospective randomised study of local anaesthetic injection after multiple rubber band ligation of haemorrhoids	2004	English	Turkey	Non-placebo	1-2mL 0.5% bupivacaine with 1:200000 epinephrine injected proximal to band after RBL	Participants blinded, investigators not specified	142 (70, 72)	10 days	10-point visual analogue scale
Hooker	Local Injection of Bupivacaine After Rubber Band Ligation of Hemorrhoids	1999	English	Canada	Non-placebo and placebo (saline injection)	0.5mL 0.5% bupivacaine with 1:200,000 epinephrine injected post RBL	Double-blind	115 (42, 31 non-placebo; 42 placebo)	1 week	100-point linear analogue scale
Kwok	The use of local anaesthesia in haemorrhoidal banding: a randomized controlled trial	2013	English	New Zealand	Non-placebo	1mL 0.5% bupivacaine, injected just proximal to band	Single-blind	72 (40, 32)	24 hours	10-point visual analogue scale
Law	Triple rubber band ligation for haemorrhoids: Prospective, randomized trial of use of local anesthetic injection	1999	English	Hong Kong	Non-placebo	1-2mL 2% lignocaine injected into banded tissue after RBL	Investigators blinded, participants not specified	101 (62, 39)	6 weeks	4-point categorical scale
Sharma	The use of topical anaesthetic in the banding of internal haemorrhodis: a feasability study for a randomised controlled trial	2022	English	United Kingdom	Non-placebo	11mL 230mg lignocaine hydrochloride gel, applied around haemorrhoid base 5 minutes before RBL	Single-blind	35 (18, 17)	72 hours	10-point visual analogue scale
Williams	An Assessment of Anesthetic-Steroid Suppositories: A controlled trial following rubber-band ligation of haemorrhoids	1972	English	United Kingdom	Placebo (inert suppository)	PR insertion of 2 suppositories containing hydrocortisone, cinchocaine and framycetin	Double-blind	20 (crossover trial)	Not specified	Pain / no pain (binary outcome)

Note. RBL = rubber band ligation. Not all studies reported the exact timing or location of anaesthetic administration, or strength of the anaesthetic; the available information is presented.
