# Supplementary material for: Analgesic effect of local anaesthetic in haemorrhoid banding: systematic review and meta-analysis
Source: Int J Colorectal Dis. 2024 Mar 4;39(1):34. doi: 10.1007/s00384-024-04609-8 (PMC10912253; doi:10.1007/s00384-024-04609-8)
Supplement: Supplementary file 3 — Supplementary file3 (PDF 126 KB) [file 384_2024_4609_MOESM3_ESM.pdf]

Journal: International Journal of Colorectal Disease

Article title: Analgesic effect of local anaesthetic in haemorrhoid banding: systematic review and meta-analysis

Authors: Eleanor G R Watson[1*], Hwa Ian Ong[2], Nicholas J W Shearer[3], Philip J Smart[2], Adele N Burgess[2], David M Proud[2], Helen M Mohan[2]

[1]University of Melbourne (Faculty of Medicine, Dentistry and Health Sciences), Melbourne (VIC), Australia.

[2]Austin Hospital (Department of Surgery), Melbourne (VIC), Australia.

[3]Royal Melbourne Hospital (Department of Anaesthesia), Melbourne (VIC), Australia.

*Corresponding author

E-mail: egwatson@student.unimelb.edu.au

ORCID: 0000-0003-3614-5132

Twitter: @dreleanorwatson

Population: patients undergoing haemorrhoid banding
Intervention: local anaesthetic
Comparison: no local anaesthetic
Outcome: post-procedural pain

Certainty assessment							№ of patients		Effect	Certainty	Importance
№ of studies	Study design	Risk of bias	Inconsistency	Indirectness	Imprecision	Other considerations	local anaesthetic	no local anaesthetic	Absolute (95% CI)		
**Pain at 1 hour (Scale from: 0 to 10)**											
5	randomised trials	not serious	serious[a]	not serious	serious[b]	none	175	167	MD **1.43 lower** (2.3 lower to 0.56 lower)	⨁⨁◯◯ Low	CRITICAL
**Pain at 6 hours (Scale from: 0 to 10)**											
3	randomised trials	not serious	serious[a]	not serious	very serious[b]	none	130	120	MD **0.52 lower** (1.04 lower to 0.01 higher)	⨁⨁◯◯ Low	CRITICAL
**Pain at 24 hours (Scale from: 0 to 10)**											
3	randomised trials	not serious	serious[a]	not serious	very serious[b]	none	127	120	MD **0.31 lower** (0.82 lower to 0.19 higher)	⨁⨁◯◯ Low	CRITICAL

Note. CI = confidence interval; MD = mean difference.

Explanations
a. at one hour I^2 was 74% (50 – 90%), and at six and 24 hours I^2 was measured as 0% due to the difference crossing zero.
b. wide confidence intervals, which crossed zero at six and 24 hours, and small sample sizes.
