# Supplementary material for: Analgesic effect of local anaesthetic in haemorrhoid banding: systematic review and meta-analysis
Source: Int J Colorectal Dis. 2024 Mar 4;39(1):34. doi: 10.1007/s00384-024-04609-8 (PMC10912253; doi:10.1007/s00384-024-04609-8)
Supplement: Supplementary file 4 — Supplementary file4 (PDF 81 KB) [file 384_2024_4609_MOESM4_ESM.pdf]

Journal: International Journal of Colorectal Disease

Article title: Analgesic effect of local anaesthetic in haemorrhoid banding: systematic review and meta-analysis

Authors: Eleanor G R Watson[1*], Hwa Ian Ong[2], Nicholas J W Shearer[3], Philip J Smart[2], Adele N Burgess[2], David M Proud[2], Helen M Mohan[2]

[1]University of Melbourne (Faculty of Medicine, Dentistry and Health Sciences), Melbourne (VIC), Australia.

[2]Austin Hospital (Department of Surgery), Melbourne (VIC), Australia.

[3]Royal Melbourne Hospital (Department of Anaesthesia), Melbourne (VIC), Australia.

*Corresponding author

E-mail: egwatson@student.unimelb.edu.au

ORCID: 0000-0003-3614-5132

Twitter: @dreleanorwatson

(a)

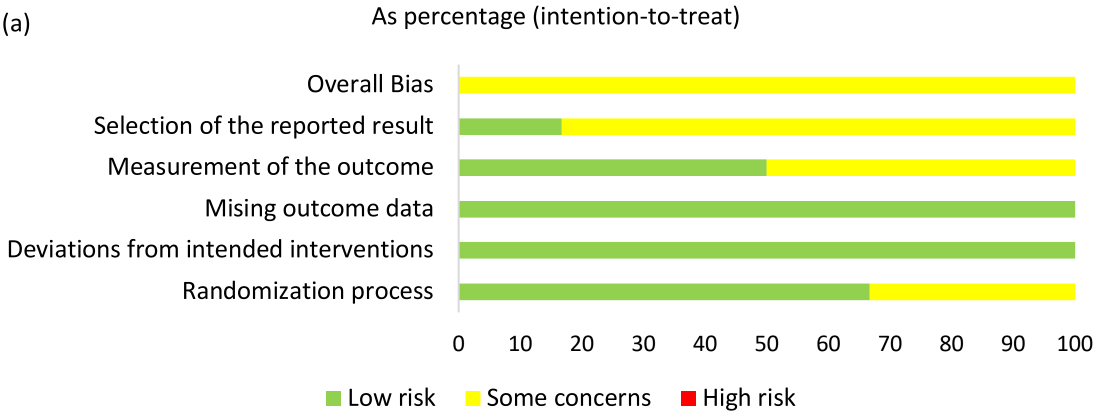

As percentage (intention-to-treat)
Overall Bias
Selection of the reported result
Measurement of the outcome
Mising outcome data
Deviations from intended interventions
Randomization process
0
10
20
30
40
50
60
70
80
90
100
Low risk
Some concerns
High risk

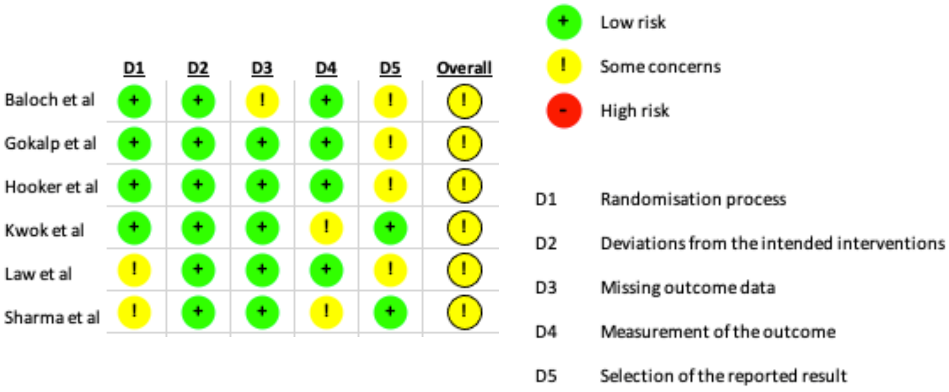

D1
D2
D3
D4
D5
Overall
Baloch et al
Gokalp et al
Hooker et al
Kwok et al
Law et al
Sharma et al
Low risk
Some concerns
High risk
D1 Randomisation process
D2 Deviations from the intended interventions
D3 Missing outcome data
D4 Measurement of the outcome
D5 Selection of the reported result

(b)

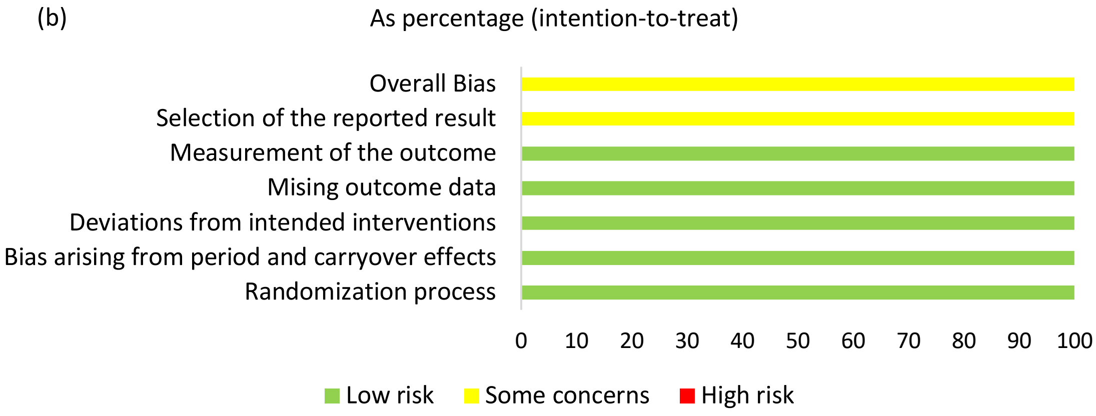

As percentage (intention-to-treat)
Overall Bias
Selection of the reported result
Measurement of the outcome
Mising outcome data
Deviations from intended interventions
Bias arising from period and carryover effects
Randomization process
0
10
20
30
40
50
60
70
80
90
100
Low risk
Some concerns
High risk
